# Genotypically Identifying Wheat Mesophyll Conductance Regulation under Progressive Drought Stress

**DOI:** 10.3389/fpls.2016.01111

**Published:** 2016-08-08

**Authors:** Katarina Olsovska, Marek Kovar, Marian Brestic, Marek Zivcak, Pavol Slamka, Hong Bo Shao

**Affiliations:** ^1^Jiangsu Key Laboratory for Bioresources of Saline Soils, Provincial Key Laboratory of Agrobiology, Institute of Agro-biotechnology, Jiangsu Academy of Agricultural SciencesNanjing, China; ^2^Department of Plant Physiology, Faculty of Agrobiology and Food Resources, Slovak University of Agriculture in NitraNitra, Slovakia; ^3^Department of Agrochemistry and Plant Nutrition, Faculty of Agrobiology and Food Resources, Slovak University of Agriculture in NitraNitra, Slovakia; ^4^Yantai Institute of Coastal Zone Research, Chinese Academy of SciencesYantai, China

**Keywords:** photosynthesis, drought, mesophyll conductance, A_N_/C_i_, carboxylation efficiency, wheat

## Abstract

Photosynthesis limitation by CO_2_ flow constraints from sub-stomatal cavities to carboxylation sites in chloroplasts under drought stress conditions is, at least in some plant species or crops not fully understood, yet. Leaf mesophyll conductance for CO_2_ (g_m_) may considerably affect both photosynthesis and water use efficiency (WUE) in plants under drought conditions. The aim of our study was to detect the responses of g_m_ in leaves of four winter wheat (*Triticum aestivum* L.) genotypes from different origins under long-term progressive drought. Based on the measurement of gas-exchange parameters the variability of genotypic responses was analyzed at stomatal (stomata closure) and non-stomatal (diffusional and biochemical) limits of net CO_2_ assimilation rate (A_N_). In general, progressive drought caused an increasing leaf diffusion resistance against CO_2_ flow leading to the decrease of A_N_, g_m_ and stomatal conductance (g_s_), respectively. Reduction of g_m_ also led to inhibition of carboxylation efficiency (Vc_max_). On the basis of achieved results a strong positive relationship between g_m_ and g_s_ was found out indicating a co-regulation and mutual independence of the relationship under the drought conditions. In severely stressed plants, the stomatal limitation of the CO_2_ assimilation rate was progressively increased, but to a less extent in comparison to g_m_, while a non-stomatal limitation became more dominant due to the prolonged drought. Mesophyll conductance (g_m_) seems to be a suitable mechanism and parameter for selection of improved diffusional properties and photosynthetic carbon assimilation in C_3_ plants, thus explaining their better photosynthetic performance at a whole plant level during periods of drought.

## Introduction

At the global level, drought accompanied by low water availability in soils is considered the main environmental factor that limits plant growth and yield (Chaves et al., 2003; Nemani et al., 2003; Zhao et al., 2011). This combination may negatively affect the productivity of agricultural crops as well as natural ecosystems and the diversity of plant species (Zivcak et al., 2013). There are some strategies aimed at maintaining water resources in soils and plants, e.g., improvement of crop water use efficiency (WUE; Wang et al., 2002; Condon et al., 2004) and photosynthesis itself, which may increase crop yields in the near future (Parry et al., 2002; Flexas et al., 2013).

A water deficit develops in plants when water losses by evapotranspiration are inadequately replaced by the water flow from soil. In a natural environment, a water deficit occurs progressively from a week to months, depending upon the characteristics of the soil where the plants are grown (Cano et al., 2014). Water deficiency triggers many responses at different levels (molecular to whole plant) of plants in conditions of water scarcity (Shao et al., 2009; Zivcak et al., 2014) that involve different survival strategies (such as stress escape, avoidance or tolerance), adaptive changes and deleterious effects which can all develop even in parallel (Barnabás et al., 2008). They also include the production of many biological macro- and micro-molecules, such as nucleic acids (DNA, RNA, microRNA), proteins, carbohydrates, lipids, hormones, ions, or mineral elements (Shao et al., 2006). These responses to external limiting factors can vary and are genotype- and species-related (Rampino et al., 2006), including the length, intensity and duration of water stress (Araus et al., 2002), plant age and ontogeny (Zhu et al., 2005), light and temperature (Gallé et al., 2009), intensity of previous stresses (Flexas et al., 2009), as well the application of successive drought and recovery cycles (Gallé et al., 2011). Moreover, under natural conditions, plants are often exposed to multiple stress factors that influence photosynthesis and growth (Lu et al., 2003). The combination of drought with other abiotic stress factors, such as intense light, salinity or heat, considerably increases the photoinhibition of photosynthesis (Shao et al., 2006; Yan et al., 2013).

The impact of drought on photosynthesis can basically be divided into two groups: (i) a direct effect, which increases the restriction of the CO_2_ diffusion pathway via stomata, as intercellular airspaces leading to the mesophyll cells that cause a decline in CO_2_ availability for Rubisco (Cornic et al., 1989; Chaves, 1991; Flexas et al., 2004a,b, 2007; McDowell, 2011), (ii) an indirect effect, such as alterations in the biochemistry and metabolism of the photosynthetic apparatus, membrane permeability (aquaporins) (Lawlor and Cornic, 2002; Chaves et al., 2009) and the promotion of oxidative stress (Aranda et al., 2012).

Indeed, restricted CO_2_ diffusion from the atmosphere to the site of carboxylation is the main reason for decreased photosynthesis under water stress conditions caused by both the stomatal and mesophyll limitations (Centritto et al., 2003; Flexas et al., 2004a,b; Grassi and Magnani, 2005; Zivcak et al., 2014). Stomata are the primary component of the CO_2_ diffusional pathway, which limits water loss. Under prolonged drought, they also limit the CO_2_ supply inside the leaves (Martorell et al., 2014). In C_3_ plants, low g_s_ reduces water loss from drying plants to save water via a rapid and effective survival strategy. The stomata response could vary in degree, becoming more pronounced with the increasing severity of a stress (Zivcak et al., 2013). The net CO_2_ assimilation rate (A_N_) is usually reduced by water deficit due to not only stomatal closure but also non-stomatal processes (Medrano et al., 2002) such as decreased g_m_ (Flexas et al., 2008). According to Fick's first law of diffusion, A_N_ is determined as follows: A_N_ = g_s_·(C_a_ − C_i_) = g_m_·(C_i_−C_c_), where C_a_, C_i_, and C_c_ are the CO_2_ concentrations in the atmosphere, sub-stomatal cavities and carboxylation site of Rubisco, respectively (Long and Bernacchi, 2003). Previous works usually stated that g_m_ is large and constant (therefore, C_i_ = C_c_). However, at present, there are many lines of evidence suggesting that the CO_2_ concentration in chloroplasts is significantly lower than in sub-stomatal cavities because of the finite value of g_m_ (von Caemmerer and Evans, 1991; Niinemets et al., 2009). Although g_m_ is rather small, it markedly regulates C_*c*_ and hence limits leaf photosynthesis (Di Marco et al., 1990; Harley et al., 1992; Loreto et al., 1992; Warren and Adams, 2006).

The mesophyll conductance indicates the conductance for CO_2_ flowing from the intercellular air spaces to the site of carboxylation in the chloroplasts of mesophyll cells and includes the quite complicated pathways of the cell wall, plasma membrane, chloroplast envelope, and stromal thylakoids. It involves gas phase resistance among intercellular air spaces and liquid phase resistance from the cell wall to stroma (Evans et al., 2009). Recent studies show a crucial role for g_m_ in the regulation of photosynthesis, and it has already been assumed that g_m_ represents up to 40% of the CO_2_ diffusional limitations to whole photosynthesis (Warren, 2008).

Currently, there are many studies showing decreased g_m_ during a progressive leaf water deficit. Recent studies (Roupsard et al., 1996; Flexas et al., 2004a, 2006; Delfine et al., 2005; Galmés et al., 2007, 2011; Tomás et al., 2013; Niinemets and Keenan, 2014) clearly confirm that drought in plants may significantly limit g_m_. Nevertheless, it remains unknown which mechanisms are responsible for the reduction of g_m_. Any changes in g_m_ during low soil water availability may potentially play an important role in the regulation and control of photosynthesis (Flexas et al., 2014). It is hypothesized that a crop under drought stress should reach low stomatal conductance (g_s_), which can reduce water loss but consequently maintains a high intensity of carbon fixation. This is only possible when the CO_2_ concentration in chloroplasts (C_c_) remains high as a result of improved g_m_ (Flexas et al., 2012).

The high sensitivity of g_m_ to different environmental factors has already been shown with the reactions occurring in a wide time range, from minutes to hours (Pons and Welchen, 2003; Flexas et al., 2012). Recent reviews have already highlighted the effects of environmental conditions, such as increased and decreased CO_2_ concentration around leaves (Harley et al., 1992; Centritto et al., 2003), exogenous application of ABA and polyethyleneglycol (Flexas et al., 2006), high altitude (Vitousek et al., 1990), low light (Laisk et al., 2005), low nitrogen availability (Warren and Adams, 2006), low and high temperatures (Bernacchi et al., 2002; Pons and Welchen, 2003; Yamori et al., 2006), or viral infections (Sampol et al., 2003). There is also increasing evidence to suggest a significant role for aquaporins in the control of membrane permeability to CO_2_, which are also limiting factors of g_m_ in C_3_ plants (Heckwolf et al., 2011; Sade et al., 2014). In particular, g_m_ is also determined by the variability of leaf structural traits, such as leaf thickness, cell packing, shape, and wall thickness (Tosens et al., 2012; Tomás et al., 2013; Muir et al., 2014).

The decrease in A_*N*_ as a consequence of water stress is also commonly analyzed in terms of the stomatal and non-stomatal limitations (Grassi and Magnani, 2005). However, the dynamics between the stomatal and non-stomatal limitations during drought remain unclear (Lawlor and Cornic, 2002; Loreto and Centritto, 2008). In previous decades, valuable studies of sufficient quantity accumulated on the effect of drought on g_m_. Indeed, inter-specific genotypic differences in g_m_ have already been found for several species, e.g., *Vitis vinifera* (Tomás et al., 2013), *Hordeum vulgare* (Barbour et al., 2010), *Castanea sativa, Solanum lycopersicum* (Galmés et al., 2011), *Oryza sativa* (Gu et al., 2012), and *Triticum aestivum* (Jahan et al., 2014).

The aim of this work was to perform an eco-physiological analysis of the main diffusional limits to leaf photosynthesis in wheat under a long-term progressive drought by determination of the dynamics and proportion of mesophyll vs. stomatal limitation changes and their sensitivity to water scarcity in four winter wheat genotypes of different geographical proveniences.

## Materials and methods

### Biological material and cultivation

**Figure 1:**
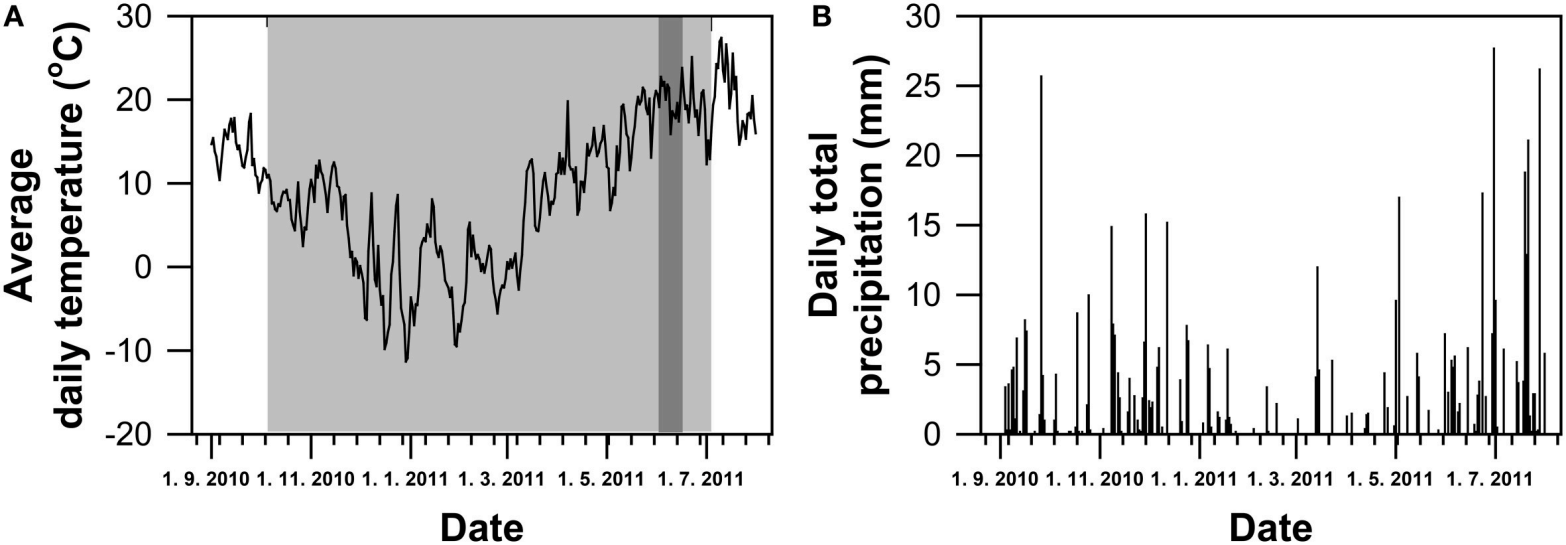
**Climatic variables (average daily temperature—(A) and daily total precipitation—(B)) during the grown period of winter wheat**. Gray and dark-gray areas represents time of cultivation period and dehydration, respectively.

The outdoor pot experiment was conducted in the experimental cage of the Department of Plant Physiology, Slovak University of Agriculture in Nitra. Seeds of four winter wheat (*T. aestivum* L.) genotypes (Šamorínska from Slovakia, GK Forrás from Hungary, Pehlivan from Turkey and Piopio-4 from Mexico) were selected on the basis of their (i) geographical origin (European genotypes–Middle to South Europe vs. Latin America), (ii) historical view of wheat breeding (Šamorínska as a landrace vs. GK Forrás, Pehlivan, and Piopio-4 as modern genotypes) and (iii) different mechanism of WUE regulation under drought conditions. They were obtained from the Gene bank in Plant Production Research Institute in Piestany (Slovakia). The seeds were sown in plastic pots (15 l volume) filled with a mixture of horticultural substrate and clay soil in 1:1 ratio. The substrate of pH 7.3 contained 40.08 mg kg^−1^ N_an_, 206.5 mg kg^−1^ P, 590 mg kg^−1^ K, and 3.73% of humus. Plants were grown in a natural environmental conditions and were regularly irrigated to maintain the optimum field water capacity during whole experiment. The foliar application of liquid fertilizers with macro- and micro-nutrients was carried out in the early spring time. At the growth stage of inflorescence emergence (BBCH-51, Zadoks et al., 1974), the progressive dehydration of soil and plants in pots was induced by a withholding watering for 21 days. The responses of photosynthesis and water status to the induced water stress were measured simultaneously from gas exchange and leaf RWC data. The leaf hydration range was used for differentiation of the water stress level, and the data were clustered into three groups, e.g., well-watered plants (WW; RWC = 80–100%), mild water stress (MS; RWC = 60–80%) and severe (SS; RWC = 40–60%) water stress. After the dehydration period watering of plants continued optimally. Climatic data (average daily temperature and daily total precipitation; Figure 1) were obtained from the meteorological station of Horticulture and Landscape Engineering Faculty in SUA Nitra, localized in neighborhood of the experimental site.

### Gas exchange and chlorophyll *a* fluorescence measurements

Gas exchange measurements were made daily on fully expanded flag leaves of control and stressed plants from the beginning of the dehydration process to its terminal phase when the stomata were fully closed.

The A_N_/C_i_ response curves of plants from each genotype were measured on a daily basis using the open gas-exchange system Li-6400XT (Li-Cor Inc., Lincoln, Nebraska, USA) with an integrated fluorescence chamber head Li-6400-40 (Li-Cor Inc.). Gas-exchange and chlorophyll *a* fluorescence parameters were measured in light-adapted leaves at saturation PPFD set up at 1500 μmol m^−2^ s^−1^ with 10% blue light to maximize stomatal aperture. Leaf temperature was kept at 21°C and relative air humidity was maintained between 60 and 70% during all measurements. Gas exchange and chlorophyll *a* fluorescence were first measured after reaching steady-state at 380 μmol CO_2_ mol^−1^ air surrounding the leaf (C_a_). Subsequently, C_a_ was decreased stepwise until 50 μmol mol^−1^ and then increased stepwise until 1500 μmol CO_2_ mol^−1^. The number of different C_a_ values used for the A_N_/C_i_ response curves was 12, and the time between the two consecutive measurements at different C_a_ values was maximal 4 min.

The actual photochemical efficiency of photosystem II (Φ_PSII_) was assessed following the procedures of Genty et al. (1989) based on the measurements of actual (F_s_) and maximal (F${}_{\text{m}}^{\prime }$) fluorescence during pulse light saturation (intensity 8000 μmol m^−2^ s^−1^) and calculated as follows:

$$\Phi_{\text{PSII}}=\left({\text{F}}_{\text{m}}{}^{\prime }-\;{\text{F}}_{\text{s}}\right)/{\text{F}}_{\text{m}}{}^{\prime }$$

The electron transport rate (J_f_) was calculated as:

$$\begin{array}{l} {\text{J}}_{\text{f}}=\Phi_{\text{PSII}}\cdot \text{PPFD}\cdot \alpha \cdot \beta \end{array}$$

where PPFD is the photosynthetically active photon flux density, α is leaf absorbance (0.85), and β is the partitioning of absorbed quanta between the PSII and PSI. The method of Valentini et al. (1995) was used to determine the product of α.β from the relationship between Φ_PSII_ and Φ_CO2_ (Φ_CO2_ = (A_N_ + R_d_)/PPFD), where R_d_ is the daytime respiration rate determined by the Laisk method (Laisk, 1977) (see next section) obtained by varying C_a_ (11 different values) under non-photorespiratory conditions in an atmosphere containing less than 1% O_2_, a leaf temperature of 21°C, saturation PPFD (1500 μmol m^−2^ s^−1^) and a relative humidity of 75%.

Flow of CO_2_ out and into the leaf cuvette was determined for the range of C_a_ values used with photosynthetically inactive leaves (obtained by heating) of each genotype enclosed in the chamber; the correction was used for the calculation of CO_2_ fluxes (Flexas et al., 2007).

Leaf-intrinsic WUE_i_ was calculated as A_N_ to g_s_ ratio from gas-exchange measurements of C_a_ at 380 μmol CO_2_ mol^−1^ air and saturating light.

### Calculation of g_m_

The mesophyll conductance for CO_2_ (g_m_) was estimated from simultaneously measured gas-exchange and chlorophyll *a* fluorescence parameters of varying C_a_ according to Harley et al. (1992):

$$\begin{array}{l} {\text{g}}_{\text{m}}\;=\;\frac{{\text{A}}_{\text{N}}}{{\text{C}}_{\text{i}}\;-\;\frac{\Gamma^{*}\cdot \left[{\text{J}}_{\text{f}}\;+\;8\cdot \left({\text{A}}_{\text{N}}\;+\;{\text{R}}_{\text{d}}\right)\right]}{{\text{J}}_{\text{f}}\;-\;4\cdot \left({\text{A}}_{\text{N}}\;+\;{\text{R}}_{\text{d}}\right)}} \end{array}$$

where A_N_, J_f_ and C_i_ were obtained during the dehydration from gas-exchange measurements of C_a_ at 380 μmol CO_2_ mol^−1^ air and saturating light. The chloroplastic CO_2_ compensation point (Γ^*^) and daytime respiration rate (R_d_) were estimated using the method of Laisk (1977). Several A_N_/C_i_ response curves were measured at three different PPFDs (50, 150, and 300 μmol m^−2^ s^−1^) and six different C_a_ levels (from 250 to 50 μmol mol^−1^) for each genotype in well-watered plants. The intersection point of the linear regression of A_N_/C_i_ response curves was used to determine the apparent CO_2_ compensation point, C${}_{\text{i}}^{*}$ (*x*-axis) and R_d_ (*y*-axis). C${}_{\text{i}}^{*}$ was used as a proxy for Γ^*^ (Warren and Adams, 2006). The measured data of R_d_ and Γ^*^ which were used for the calculation of g_m_ are shown in Table 1.

**Table 1:** The CO_2_ compensation point in the absence of respiration (Γ^*^; μmol mol^−1^) and the mitochondrial respiration rate under light (R_d_; μmol m^−2^ s^−1^) as measured in four wheat genotypes under well-watered conditions.

	Γ^*^ (μmol mol^−1^)	**R_d_ (μmol m^−2^ s^−1^)**
GK Forrás	36.38 ± 2.58	2.18 ± 0.07
Pehlivan	34.86 ± 2.61	2.13 ± 0.05
Piopio-4	35.15 ± 1.01	2.14 ± 0.06
Šamorínska	34.08 ± 1.70	2.08 ± 0.04

*Data represent the means of set measurements performed by the Laisk method ± S.E. (n = 3)*.

### Calculation of Vc_max_

The maximal *in vivo* carboxylation activity of Rubisco (Vc_max_) was calculated from the gas exchange measurement by the data fitting procedure of the initial slope of the A_N_/C_i_ curve (C_i_ < 300 μmol mol^−1^):

$$\begin{array}{l} \text{A}=\frac{{\text{Vc}}_{\text{max}}\cdot \left({\text{C}}_{\text{i}}\;-\;\Gamma^{*}\right)}{{\text{C}}_{\text{i}}\;+\;{\text{K}}_{\text{c}}\cdot \left(1\;+\;\frac{\text{O}}{{\text{K}}_{\text{o}}}\right)} \end{array}$$

where A is the net assimilation rate limited by Rubisco activity, and K_c_ and K_o_ are the Michaelis-Menten constants of Rubisco activity for RuBP carboxylation and oxygenation, respectively. K_c_ and K_o_ are assumed to be 404.9 μmol mol^−1^ and 278.4 mmol mol^−1^ at 25°C, respectively, according to Bernacchi et al. (2001). Oxygen concentration in chloroplasts (O) was assumed to be 210 mmol mol^−1^.

### Estimation of relative limitation to photosynthesis

The limitation of photosynthesis based on g_s_ and g_m_ was estimated as potential rate of photosynthesis assuming these conductance values were infinite or measured, respectively (Farquhar and Sharkey, 1982). A_N_/C_i_ curves were used to separate and estimate the stomatal and non-stomatal limitations to photosynthesis. To assess an effect of dehydration on CO_2_ assimilation, the photosynthetic limitations were partitioned into the components related to stomatal and mesophyll conductance according to Warren et al. (2003) and calculated as follows:

$$\begin{array}{lll} {\text{L}}_{\text{S}} & = & 100\cdot \frac{{\text{A}}_{\text{Ci}}\;-\;{\text{A}}_{\text{Ca}}}{{\text{A}}_{\text{Ci}}}\\ {\text{L}}_{\text{M}} & = & 100\cdot \frac{{\text{A}}_{\text{Cc}}\;-\;{\text{A}}_{\text{Ca}}}{{\text{A}}_{\text{Cc}}} \end{array}$$

where L_S_ and L_M_ are the relative stomatal and mesophyll limitation of A_N_, respectively, A_Ca_ is the light-saturated rate of photosynthesis at C_a_ = 380 μmol mol^−1^ (g_s_ and g_m_ as measured), A_Ci_ is the light-saturated rate of photosynthesis at C_i_ = 380 μmol mol^−1^ (assuming g_s_ was infinite and g_m_ was measured), and A_Cc_ is the light-saturated rate of photosynthesis at C_c_ = C_i_ (assuming g_m_ was infinite and g_s_ was measured).

### Relative water content

The leaf relative water content (RWC) was determined as:

$$\begin{array}{l} \text{RWC }=\;\frac{\left(\text{FW }-\text{ DW}\right)}{\left(\text{TW }-\text{ DW}\right)}\cdot 100 \end{array}$$

The leaf disc was cut out from the central part of a measured leaf. Fresh weight (FW) was determined immediately after the gas exchange measurement. Turgid weight (TW) was obtained after 12 h of hydration, when a leaf disc was kept in distilled water at 4°C in the dark. Dry weight (DW) was measured after drying the leaf disc at 80°C for 24 h.

### Statistical analyses

The experiment with wheat plants in pots was established by block method with a completely randomized design of experimental plots. All analyses were performed using the Statistica v. 10 software (StatSoft Inc., Tulsa, Oklahoma, USA) and the graphics software SigmaPlot version 11.0 (Systat Software Inc., San Jose, California, USA). Analysis of variance was performed between the different levels of drought (well-watered, mild and severe water stress) at a significance level of 0.05, and Duncan's *post hoc* test was used. The variability between investigated genotypes was tested by the HSD test.

## Results

Climatic conditions at the experimental site are shown in Figure 1. Average daily temperature during the growing season (October 5, 2010 to July 4, 2011) was 7.4°C with the sum of precipitation of 373.9 mm. The sum of active daily temperatures (above 10°C) per growing season was 1658°C. The average daily temperature during the drought treatment was 20.03°C.

Significant differences among the investigated wheat genotypes grown in WW conditions were found for A_N_, g_s_, g_m_, and Vc_max_. The A_*N*_ and g_s_ varied from 26.39 to 28.64 μmol m^−2^ s^−1^ and 0.50 to 0.43 mol m^−2^ s^−1^, respectively. Differences among wheat genotypes for WUEi were non-significant (*p* > 0.05) and varied from 56.87 to 64.52 μmol CO_2_ mol^−1^ H_2_O. Genotype Pehlivan reached the highest value for these parameters (Table 2). Genotypic variation in g_s_ (c.v. 12%) explained 7% of the observed variability in A_N_ under WW conditions (Table 3). Mesophyll conductance (g_m_) in WW plants varied nearly 3-fold among all genotypes, from 0.24 to 0.73 mol m^−2^ s^−1^ (*p* < 0.001). The highest value for g_m_ was observed in the genotype Pehlivan.

**Table 2:** **The net CO_2_ assimilation rate (A_N_; μmol m^−2^ s^−1^), stomatal conductance to H_2_O (g_s_; mol H_2_O m^−2^ s^−1^), mesophyll conductance to CO_2_ (g_m_; mol CO_2_ m^−2^ s^−1^) and leaf-intrinsic water use efficiency (WUE^i^ calculated as A_N_/g_s_ ratio; μmol CO_2_ mol^−1^ H_2_O) in flag leaves of four wheat genotypes under well-watered (WW; RWC = 80–100%), mild stressed (MS; RWC = 60–80%) and severe stressed (SS; RWC = 40–60%) conditions**.

		**A_N_**	**g_s_**	**g_m_**	**WUE**
GK Forrás	WW	27.61 ± 1.67^Aa^	0.43 ± 0.06^Ba^	0.45 ± 0.04^Ba^	64.52 ± 8.45^Ab^
	MS	16.01 ± 2.25^Bb^	0.39 ± 0.08^ABb^	0.16 ± 0.06^Ab^	42.18 ± 5.51^Bc^
	SS	7.12 ± 2.11^ABc^	0.09 ± 0.06^Bc^	0.06 ± 0.02^Ac^	124.22 ± 30.79^Aa^
Pehlivan	WW	28.64 ± 1.82^Aa^	0.50 ± 0.04^Aa^	0.73 ± 0.09^Aa^	56.87 ± 6.32^Aa^
	MS	19.35 ± 3.38^Ab^	0.42 ± 0.04^Ab^	0.16 ± 0.08^Ab^	48.63 ± 9.83^Ba^
	SS	4.98 ± 2.19^Cc^	0.15 ± 0.04^Ac^	0.06 ± 0.03^Ac^	52.61 ± 18.67^Ba^
Piopio-4	WW	25.85 ± 1.74^Ba^	0.46 ± 0.06^Ba^	0.24 ± 0.03^Ca^	57.00 ± 3.79^Aa^
	MS	16.16 ± 2.25^Bb^	0.37 ± 0.04^Bb^	0.09 ± 0.02^Bb^	46.26 ± 9.01^Ba^
	SS	5.65 ± 2.32^BCc^	0.11 ± 0.08^ABc^	0.05 ± 0.01^Ac^	33.88 ± 6.45^Cb^
Šamorínska	WW	26.39 ± 1.10^Ba^	0.45 ± 0.06^Ba^	0.44 ± 0.07^Ba^	58.79 ± 6.72^Aa^
	MS	17.00 ± 2.43^ABb^	0.29 ± 0.08^Cb^	0.12 ± 0.03^ABb^	64.11 ± 18.00^Aa^
	SS	8.44 ± 2.11^Ac^	0.13 ± 0.04^ABc^	0.06 ± 0.01^Ac^	61.42 ± 11.97^Ba^

*The data are the means ± S.E. (n = 12–20)*.

*S.E.–standard error. superscript: large letters (A,B,C) denote significant differences at p < 0.05 obtained by Duncan's post hoc test among all wheat genotypes at a given stress level (WW, MS or SS), and small letters (a,b,c) indicate statistical differences among all stress levels for a given genotype*.

**Table 3:** **Genotypic variability of the net CO_2_ assimilation rate (A_N_; μmol m^−2^ s^−1^), stomatal conductance to H_2_O (g_s_; mol H_2_O m^−2^ s^−1^), and mesophyll conductance to CO_2_ (g_m_; mol CO_2_ m^−2^ s^−1^) in four wheat genotypes under well-watered (RWC = 80–100%), mild stress (RWC = 60–80%), and severe stress (RWC = 40–60%) conditions**.

		**Mean**	**S.E**.	**c.v**.	**F**	**P**
WW	A_N_	27.26	1.93	0.07	26.33	0.000
	gs	0.47	0.06	0.12	5.728	0.002
	gm	0.49	0.19	0.39	167.7	0.000
MS	A_N_	17.22	3.10	0.18	3.763	0.016
	gs	0.37	0.08	0.21	9.683	0.000
	gm	0.14	0.06	0.41	4.708	0.006
SS	A_N_	6.57	2.60	0.12	6.170	0.002
	gs	0.12	0.06	0.49	3.000	0.042
	gm	0.06	0.02	0.34	0.302	0.824

*S.E.,standard error; c.v., coefficient of variability; F, F ratio, p, probability*.

**Figure 2:**
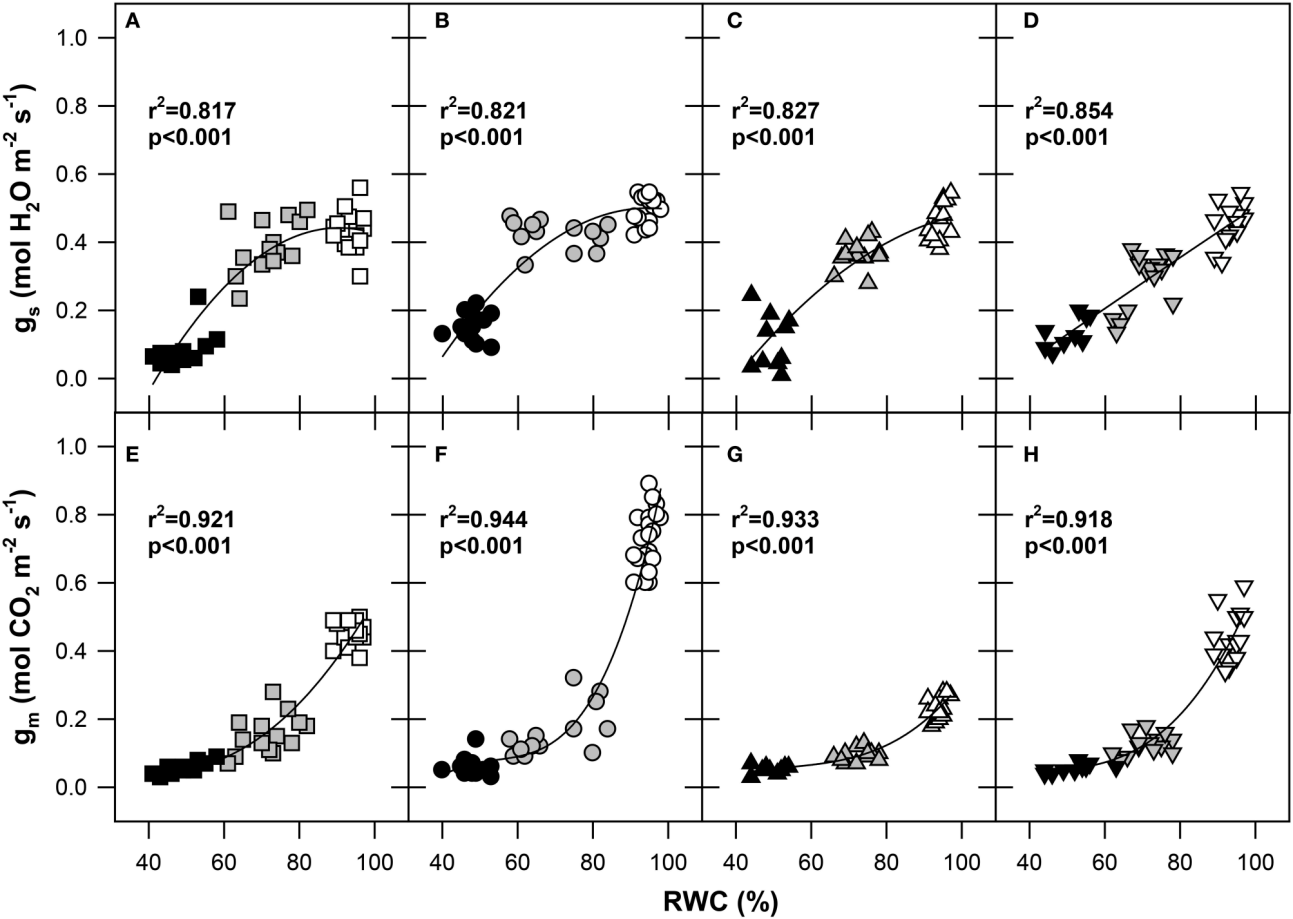
**Responses of stomatal conductance to H_2_O (g_s_; mol H_2_O m^−2^ s^−1^) and mesophyll conductance to CO_2_ (g_m_; mol CO_2_ m^−2^ s^−1^) to the relative water content (RWC; %) of flag leaves in four wheat genotypes (A, E) GK Forrás, (B, F) Pehlivan, (C, G) Piopio-4 and (D, H) Šamorínska**. The points represent individual measurements of leaves. Symbols: square-GK Forrás, circle-Pehlivan, up-triangle-Pio, down-triangle–Šamorínska; empty symbols–well-watered (RWC 80–100%) plants, gray symbols-mild stress (RWC 60–80%) and black symbols-severe stress (RWC 40–60%). The coefficients of determination (*r*^2^) and significance level (*p*) as well as the lines of polynomial quadratic **(A–D)** and polynomial cubic **(E–H)** regressions are shown.

Significant reductions in A_N_, g_s_, and g_m_ were observed under progressive dehydration from WW conditions (Figure 2, Tables 2, 3). Under the MS conditions, significant genotypic differences were found in A_N_ and g_s_, which varied from 16.01 to 19.35 μmol m^−2^ s^−1^ and 0.29 to 0.42 mol m^−2^ s^−1^, respectively. Thus, the average 1.5-fold reduction of A_N_ was accompanied by an almost 20% reduction of g_s_ and a 3.5-fold reduction of g_m_. The highest stomatal sensitivity to the decline in RWC was observed in genotype Šamorínska, while the highest sensitivity of g_m_ to RWC was found in genotype Pehlivan. Under severe water stress (SS) conditions, g_s_ declined below 0.15 mol m^−2^ s^−1^ in all genotypes, with the most pronounced reduction in GK Forrás. However, we should be noted that in genotypes Piopio-4 and Šamorínska originating from Mexico and Slovakia (Šamorínska is a Slovakian landrace), respectively, the dehydration cycle was faster (11 days), causing the g_s_ to drop below 0.08 mol m^−2^ s^−1^, while in genotypes Pehlivan and GK Forrás from Turkey and Hungary, similar g_s_ values (0.09 and 0.15 mol m^−2^ s^−1^) were reached after 15 and 16 days of dehydration, respectively. The reduction of leaf RWC resulted in the decline of g_m_ (0.05–0.06 mol m^−2^ s^−1^) with non-significant (*p* > 0.05) genotypic differences. The g_s_ and g_m_ reductions resulted in the reduction of A_N_ (Table 2). Then, the reduction of g_s_ relative to A_N_ in genotype GK Forrás under drought condition significantly (*p* < 0.001) increased WUEi. Finally, under SS conditions, the genotypic variation in g_s_ (c.v. 49%) explained 12% of the observed variability in A_N_ (Table 3).

There was a clear polynomial decline in g_s_ induced by stomatal closure in the plant response to progressive drought, showing the same trends for genotypes GK Forrás, Pehlivan, and Piopio-4 (Figures 2A–C), with the exception of genotype Šamorínska (Figure 2D), which showed almost linear decline of g_s_. This result indicates a high stomatal sensitivity of landrace genotype to water stress, confirming that stomata were completely closed after 11 days of dehydration.

**Figure 3:**
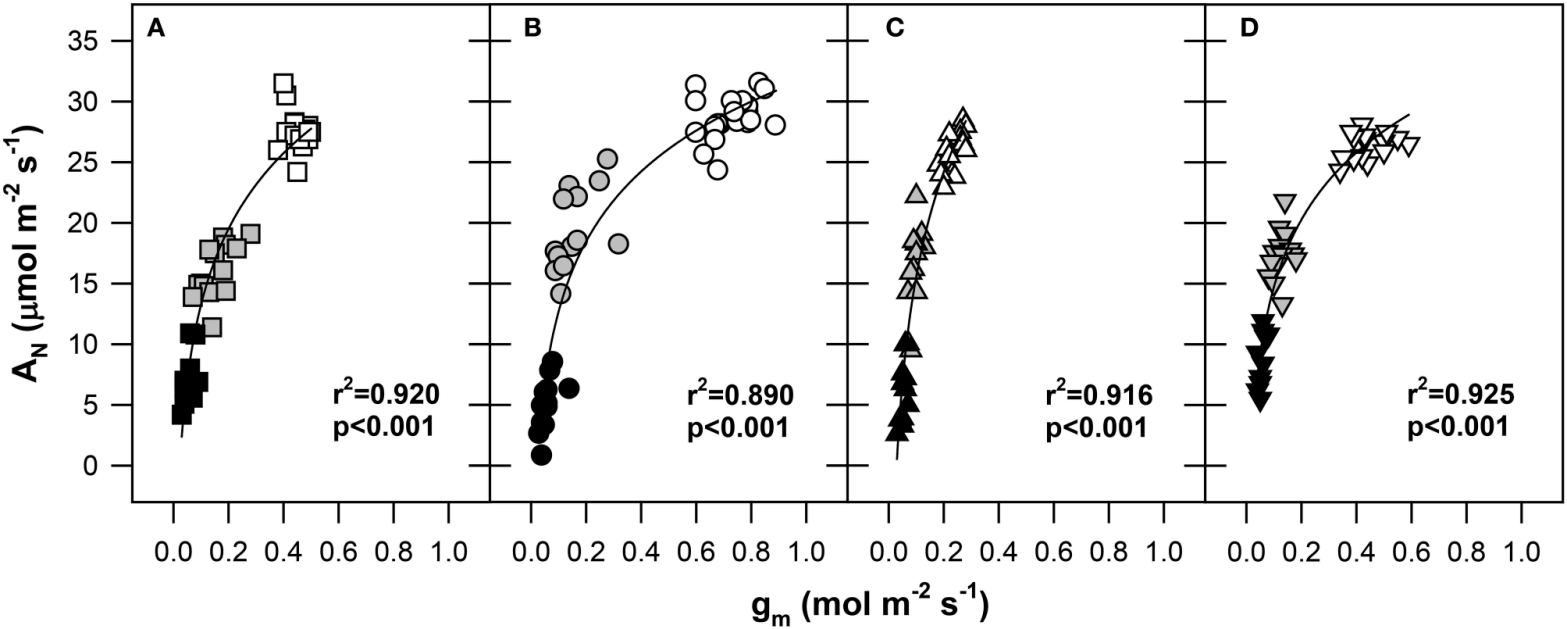
**The net CO_2_ assimilation rate (A_N_, μmol CO_2_ m^−2^ s^−1^) response to mesophyll conductance (g_m_, mol CO_2_ m^−2^ s^−1^) in (A) GK Forrás, (B) Pehlivan, (C) Piopio-4 and (D) Šamorínska genotypes under progressive drought conditions**. The points represent individual measurements of leaves. The symbols are the same as in Figure 2. The coefficients of determination (*r*^2^) and significance level (*p*) as well as the line of logarithmic regressions are shown in the plot.

As shown in Figure 3, the A_N_ was positively correlated with g_m_ under progressive dehydration in all genotypes (*r*^2^ from 0.890 for Pehlivan to 0.924 for Šamorínska; *p* < 0.001). A significant decline in A_N_ in response to reduced g_m_ was observed under the transition from WW to MS conditions. Under SS conditions, a strong reduction of g_m_ (below 0.15 mol m^−2^ s^−1^) resulted in a progressive decline of A_N_; however, this was still above the CO_2_ compensation point in all genotypes. The largest slope of the A_N_/g_m_ relationship was observed in the Piopio-4 genotype, where we conclude that the drought stress had a greater impact on g_m_ compared to A_N_.

**Figure 4:**
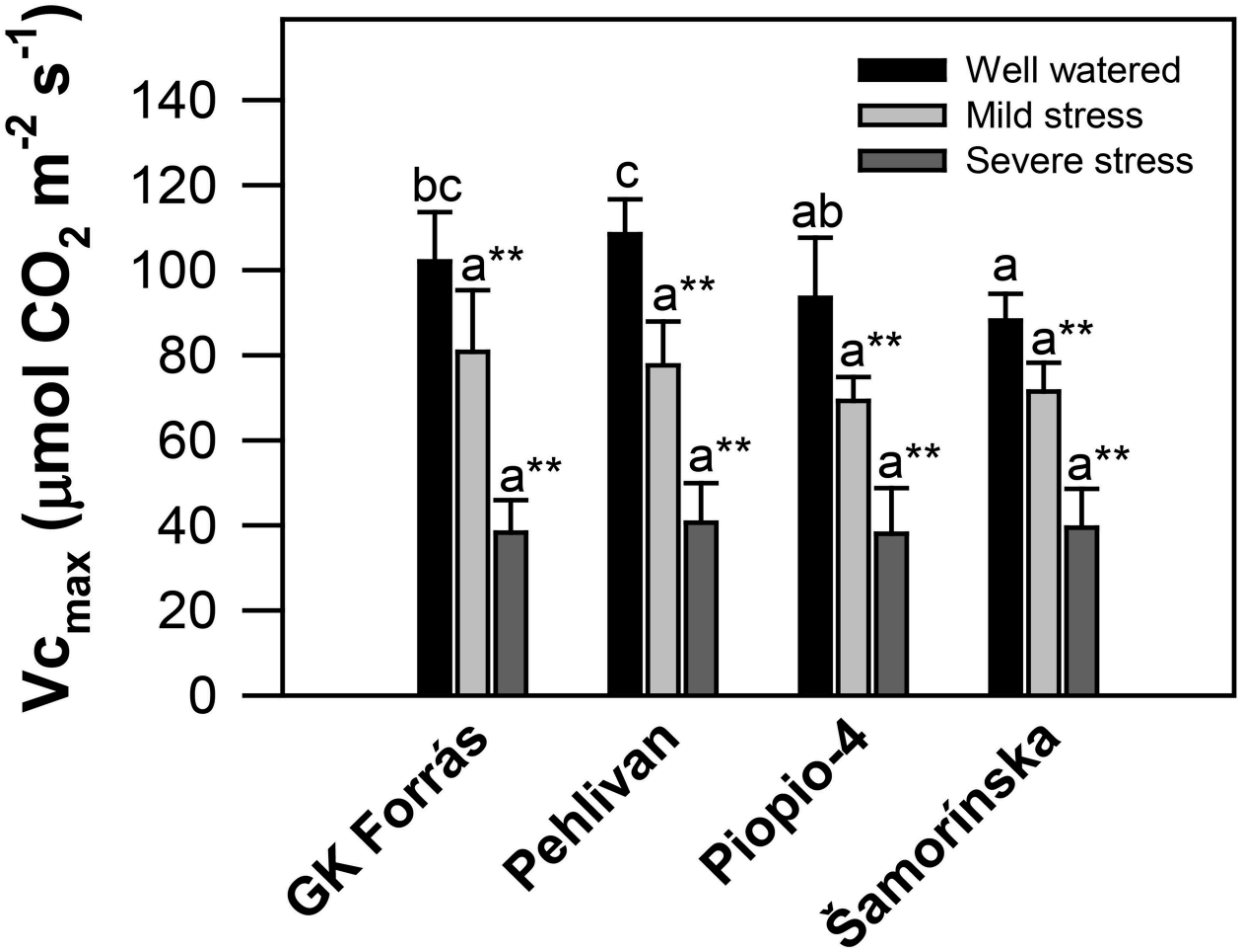
**The maximum rate of carboxylation of Rubisco (Vc_max_; μmol CO_2_ m^−2^ s^−1^) calculated from the gas-exchange data measured in the leaves of four wheat genotypes at different levels of drought stress**. Vertical bars are the means of 9–20 individual measurements per treatment ± S.E. Different letters indicate significant differences among genotypes (*p* < 0.05) based on Duncan's post hoc test at one stress level; ^**^Indicates significant differences among stress treatments (*p* < 0.01) based on the HSD test in one genotype.

Analysis of the *in vivo* maximal carboxylation activity of Rubisco (Vc_max_) revealed the genotypic variability (*p* < 0.05) only under well-watered conditions (Figure 4), with the changes ranging from 88.14 ± 6.3 to 108.44 ± 8.2 μmol m^−2^ s^−1^ for genotypes Šamorínska and Pehlivan, respectively. Water stress (MS and SS) significantly (*p* < 0.01) reduced Vc_max_, but without any genotypic difference. The mean level of Vc_max_ was 74.8 ± 5.4 and 39.12 ± 1.2 μmol m^−2^ s^−1^ both in MS and SS, which constituted ~0.5-fold and 2.5-fold decline for MS and SS, respectively.

**Figure 5:**
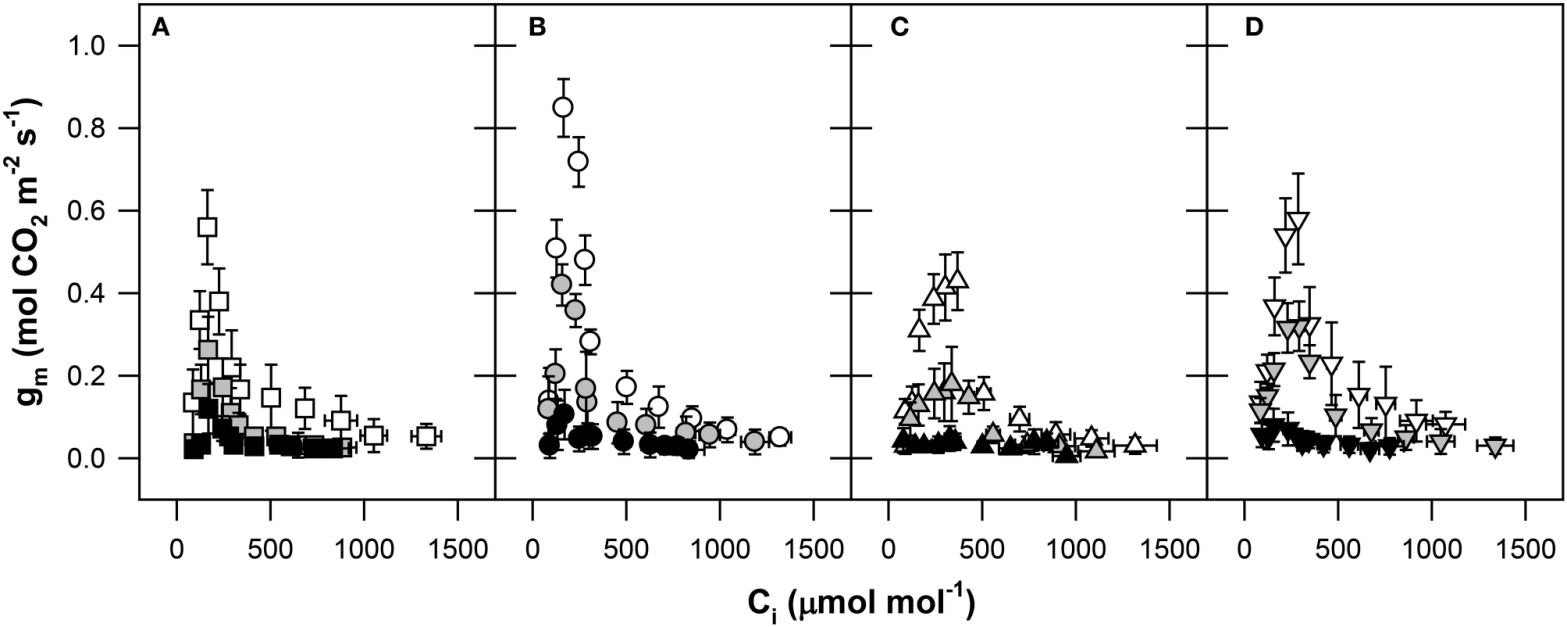
**The response of mesophyll conductance (g_m_, mol CO_2_ m^−2^ s^−1^) to rapid changes in the CO_2_ concentration within intercellular spaces (C_i_, μmol CO_2_ mol^−1^ air) in (A) GK Forrás, (B) Pehlivan, (C) Piopio-4, and (D) Šamorínska**. The points represent means from 9 to 20 individual measurements per treatment ± S.E. The symbols correspond to those presented in Figure 2.

Analysis of the fast A_N_–C_i_ response curve showed that the g_m_ calculated via the method of Harley et al. (1992) was not constant along the range of C_i_ values employed in this study (Figure 5). We observed the obviously known three-phase course of g_m_ changes to varied C_i_ values. A strong sensitivity of g_m_ at low C_i_ concentrations was observed in the first part of the response curve (C_i_ from ~80 to 200 μmol mol^−1^ air). After reaching an inflection peak of g_m_ at C_i_ concentrations from 200 to 400 μmol mol^−1^ air, the g_m_ values declined exponentially under the value of 0.1 mol m^−2^ s^−1^ at high C_i_. The maximal sensitivity of g_m_ to increased C_i_ was observed in Pehlivan (Figure 5B) with a 16-fold reduction of g_m_ observed until the steady-state level was reached. The weak sensitivity of g_m_ to increased C_i_ (only ~4-fold decline) was observed in the Piopio-4 genotype (Figure 5C). The highest genotypic differences in the sensitivity of g_m_ to C_i_ variations were observed at low C_i_ concentrations (GK Forrás and Pehlivan with relatively lower g_m_ and Piopio-4 and Šamorínska with relatively higher g_m_). Water stress reduced the sensitivity of g_m_ to C_i_ changes in all of the investigated genotypes. During the transition from the mild to severe water stress, the mechanism responsible for the g_m_ reaction was clearly inhibited, and g_m_ did not react as fast as in the case of well-watered plants. The g_m_ was negatively affected under SS conditions in all genotypes when the response to altered C_i_ was inhibited. Our results support the suggestions of others that mild to severe drought strongly influences the mechanism of g_m_ regulation (Figure 5).

**Figure 6:**
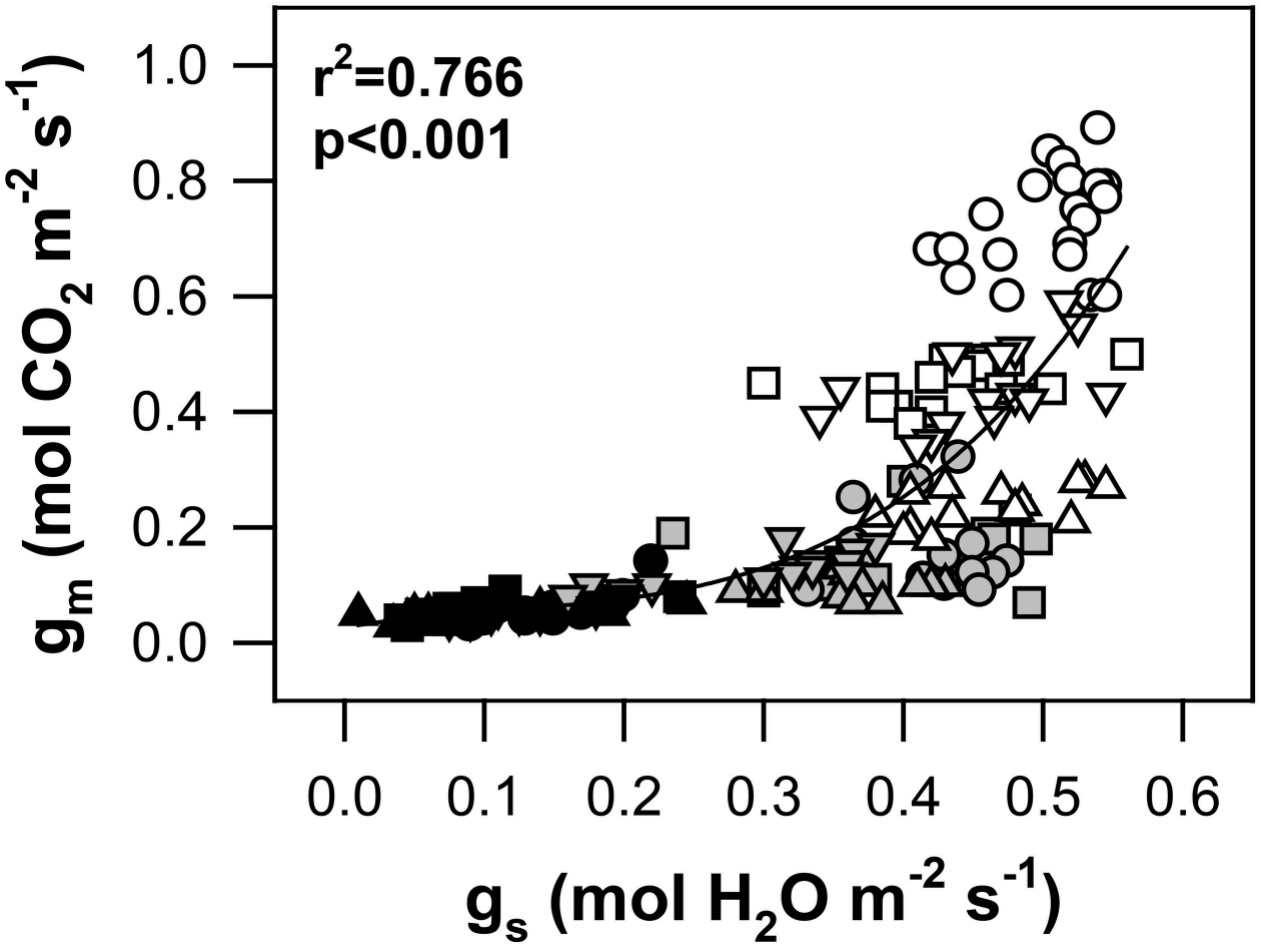
**The relationship between the stomatal conductance to H_2_O (g_s_, mol H_2_O m^−2^ s^−1^) and mesophyll conductance to CO_2_ (g_m_, mol CO_2_ m^−2^ s^−1^) in the leaves of four wheat genotypes under progressive drought**. The symbols are the same as in Figure 2. Data were fitted by a non-linear regression (full line). The equation is g_m_ = 0.426 g_s_ − 2.129 g${}_{\text{s}}^{2}\;+$ 6.189 g${}_{\text{s}}^{3}$, and the coefficient of determination (*r*^2^) and significance (*p*) are shown (*n* = 161) in the plot.

As shown in Figure 6, a close relationship between g_m_ and g_s_ was observed in all genotypes and stress levels (*r*^2^ = 0.77; *p* < 0.001). During the transition state from WW to MS conditions, the 1.5-fold reduction of g_s_ was accompanied by a 3-fold decline of g_m_. A further increase in water stress up to SS conditions resulted in progressive stomatal closure and a reduction of g_s_ accompanied by only small changes in g_m_. However, the transition from WW to MS affected both g_s_ and g_m_ in approximately the same measure. Thus, the final g_m_/g_s_ relationship was linear. The highest slope of g_m_/g_s_ was identified for genotype Pehlivan, while the lowest was identified for Piopio-4.

**Figure 7:**
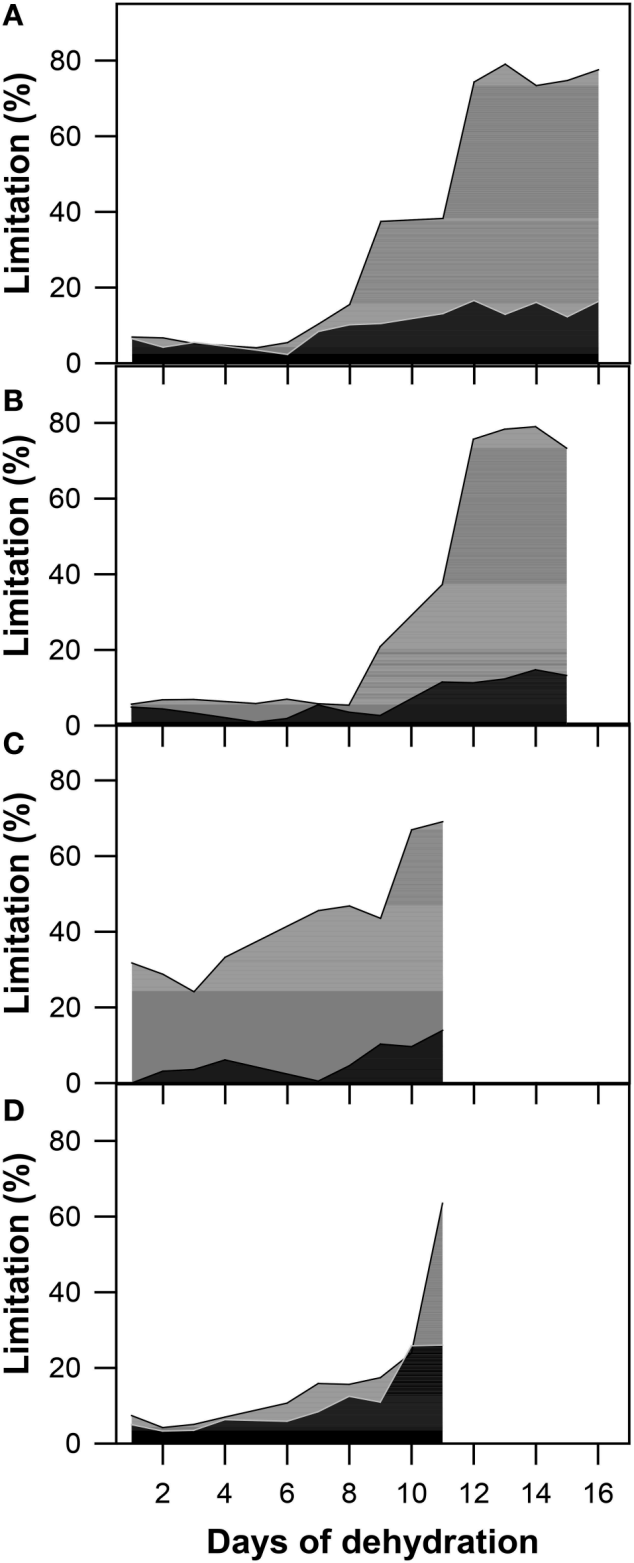
**The stomatal (L_S_; black) and mesophyll (L_M_; gray) limitations to the A_N_ calculated as the % of the maximum (well-watered) values in (A) GK Forrás, (B) Pehlivan, (C) Piopio-4, and (D) Šamorínska genotypes under progressive drought**. Each value represents the mean of four replicates.

Based on the analyses of the A_N_/C_i_ response curves measured on a daily basis during the experiment, the stomatal and mesophyll limitation ratio was calculated (Figure 7). After the determination of both limitations in all genotypes, genotypic differences in the limitations were evaluated. The observed differences could tell us more about drought response reactions and could also help determine which limitation is more crucial for the regulation of photosynthesis during drought.

From the first day of the experiment, we assessed the initial values of stomatal (L_S_) and mesophyll (L_M_) limitations as a percentage (Figure 7). As drought progressed and leaf water deficit increased, both L_S_ and L_M_ increased simultaneously, but the dynamics of the increase became uneven. L_S_ began to increase to a less extent than L_M_. The maximal value of L_S_ (22.53%) was reached in stressed plants of the old Slovak genotype Šamorínska. However, this is not a crucial value that limits leaf photosynthesis. Therefore, we suggest that L_S_ did not play as important a role in comparison with L_M_ in dehydrated plants of all selected genotypes. L_M_ predominated in three genotypes (Šamorínska, GK Forrás and Piopio-4). Although the L_S_ of Pehlivan was higher than L_M_ in the first period of dehydration, it changed after L_M_ dominated over L_S_. In genotype Piopio-4, L_M_ was mostly disabled by drought in comparison with other genotypes. It obtained very high initial values (31.91%) and increased even further with a culmination at 69.2% as the drought progressed. Additionally, a great impact of water deficit caused a significant increase in L_M_ and was found in dehydrated plants of Pehlivan (76.2%) and GK Forrás (77.6%).

## Discussion

Soil water scarcity is the main limiting factor for crop growth and yield worldwide. Despite the increased knowledge over the past decade on the effects of water stress on photosynthesis, there is still a controversial debate whether water stress limits A_N_ primarily by stomata closure (stomata limitation) or mesophyll limitation (diffusional and metabolic). A general response of plant tissues to soil water deficit is the decline of relative water content (RWC). This depends on the strength and duration of drought stress (Chaves et al., 2009). The withholding of water resulted in the reduction of stomatal conductance (g_s_) as a consequence of stomatal closure (Table 2; Figure 2) with significant genotypic differences (Table 3). The higher stomata sensitivity to RWC decline found in the genotype Šamorínska is the result of rapid water loss from leaf tissues (Figure 2D). As observed from our experimental data, modern genotypes reacted to drought by a slow reduction of g_s_ at the initial phase of dehydration, probably due to better osmotic adjustment and/or a deeper and more efficient root system (Wasson et al., 2012).

The reduction in A_N_ resulting from decreased RWC was significantly correlated with a decline in g_s_. This response is similar to those observed in many studies, and it is thought to be the general acclimation response of plants to drought (Cornic et al., 1989; Chaves, 1991; Cornic, 2000; Flexas et al., 2006). Under the gradual dehydration induced by withholding watering in plants, a highly significant relationship (*r*^2^ = 0.93; data not shown) between the RWC decline and the reduction in A_N_ was observed. Flexas et al. (2006) summarized their own results and compared them with others to reach a compromise in order to determine what limits A_N_ more, stomata closure or metabolic impairments in the mesophyll. They noted that the reduction of CO_2_ supply from the atmosphere to chloroplasts was the main factor that decreased A_N_ under drought conditions. However, metabolic impairments occurred as well, but only during stronger water stress when g_s_ dropped below 0.10 mol H_2_O m^−2^ s^−1^.

In our study with well-watered wheat plants, the observed g_m_ corresponded to the g_m_ level for wheat as found in many published works (Tazoe et al., 2009, 2011; Jahan et al., 2014; Sun et al., 2015). Interestingly, a wide interval and significant genotypic differences in g_m_ (from 0.24 to 0.73 mol m^−2^ s^−1^) (Tables 2, 3) may be the result of both the differences in Rubisco activity and the anatomical properties of leaves, respectively (Evans et al., 1994, 2009; Medrano et al., 2002; Parry et al., 2002; Flexas et al., 2006; Niinemets et al., 2009; Tomás et al., 2013; Muir et al., 2014). The role of aquaporins in the transport of CO_2_ and thus the regulation of g_m_ are also essential (Hanba et al., 2004). Inter-specific variations in g_m_ were also previously reported in a number of publications (Ethier and Livingston, 2004; Niinemets et al., 2009; Tomás et al., 2013; Niinemets and Keenan, 2014).

Based on the data analyses, a strong relationship was observed in our measurements between A_N_ and g_m_ (Figure 3). The g_m_ decreased simultaneously as A_N_ declined, which was caused by enhanced water scarcity. This trend was found for each of the studied wheat genotypes. This observed strong correlation demonstrates a well-known fact about the substantial regulation of g_m_ that is directly connected to A_N_ and thus represents the main factor underlying diffusive limitation for CO_2_ from the internal sub-stomatal cavities to the site of carboxylation (Tezara et al., 1999). Ultimately, due to this significant relationship, we could also consider g_m_ as the main factor that limits photosynthesis (Lawlor and Cornic, 2002) and plays a crucial role in the entire metabolism within the leaf mesophyll (Flexas et al., 2012).

Previously, one group of researchers argued that the decline in A_N_ occurs as a direct consequence of stomata closure, which restricts further CO_2_ diffusion from the intercellular spaces to the sites of carboxylation (Sharkey, 1990; Chaves, 1991; Cornic, 2000). On the other hand, Tezara et al. (1999) suggested that the decline of A_N_ is due to the impairment of ATP and RuBP synthesis and low ATP content, rather than stomata limitation. Another factor could be any of the processes of the Calvin cycle, although it is still not clear which of these might be involved. Moreover, drought is able to damage and influence processes involved in RuBP regeneration, e.g., activities of key enzymes of the Calvin cycle, such as fructose-1,6-bisphosphate phosphatase, NADP:glyceraldehyde-3-phosphate dehydrogenase, ribulose-5-phospho kinase, or 3-phosphoglycerate kinase (Flexas et al., 2004a).

It has been established that g_m_ is a finite variable (Niinemets et al., 2009). By simultaneously measuring gas exchange and chlorophyll *a* fluorescence, we exposed a substantial inhibition of g_m_ during the development of water stress. It has been shown that g_m_ is extremely sensitive to drought; photosynthesis in water-stressed conditions is considerably reduced (Grassi and Magnani, 2005; Flexas et al., 2006, 2007). In accordance with this, our results confirmed the differences in the kinetics of mesophyll limitation during photosynthesis (Figure 3). The genotypes Pehlivan and Piopio-4 differed the most in this regard (Figures 3B,C).

It is also well-known that g_m_ controls the metabolic and anatomical properties of leaves during photosynthesis. Both the amount and activity of Rubisco are crucial in the control of g_m_ (Niinemets et al., 2009). Therefore, we would expect a large inhibition of the maximal *in vivo* carboxylation activity of Rubisco (Vc_max_) due to prolonged dehydration, which has already been established. During mild and severe stress conditions, drought induced a significant (2.5-fold) decline in Vc_max_ in all genotypes (Figure 4). However, the Vc_max_ decline should be more pronounced than was found in our experiment. Flexas et al. (2006) achieved 94% decrease in the Vc_max_ of *Nicotiana tabacum* plants resulting from inhibition of Rubisco activity as was also confirmed by other works (Medrano et al., 1997; Parry et al., 2002). Lawlor and Tezara (2009) studied the problem of Rubisco inhibition under drought in more detail and concluded that a key for the response was a decline in the Rubisco activase enzyme activity. Similarly, Lawlor and Cornic (2002) also reported that decreased Rubisco activase activity resulted from progressive water stress.

During our experiment, the dependence between g_m_ and C_i_ was clearly demonstrated (Figure 5). Plants also differed in their g_m_ sensitivity to changing C_i_. Previously, a rapid response of g_m_ as found in our study has also been reported by Centritto et al. (2003), Flexas et al. (2007, 2014), Bunce et al. (Bunce, 2009) and Tazoe et al. (2011). However, such a deep analysis has not been presented for wheat. But, as seen from the work of Flexas et al. (2007), the relationship was established for many different plant species, such as *Arabidopsis thaliana, Limonium gibertii, N. tabacum, Vitis berlandieri* × *Vitis rupestris, Cucumis sativus*, and *Olea europaea* var. *europaea*. These studies show that g_m_ rapidly responds to changing C_i_ ranging from 50 to 1200 μmol mol^−1^ air. At high CO_2_ concentrations in sub-stomatal cavities where CO_2_ is limited by insufficient available energy, g_m_ sharply decreases.

Irrespectively to current knowledge about the function and regulation of g_m_, the mechanism leading to the photosynthetic response to varying C_i_ remains unclear. Even less is known about the intra-species variations in g_m_ at changing C_i_. It has been assumed that the genotypic divergence could be the result of different structural characteristics and features of leaves as well as the activity of membrane aquaporins (von Caemmerer and Evans, 1991; Kjellbom et al., 1999). Another possible mechanism clearly affecting g_m_, but not linked to the function of aquaporins, is chloroplast swelling and movement (Flexas et al., 2007).

Co-regulation between g_m_ and g_s_ is currently debated by many scientists. This is still a complicated question because CO_2_ diffusion from the ambient air directly into the chloroplasts is defined by g_s_ and g_m_ together, which could vary either over the long-term periods of leaf morphological changes or over short-term changes in chloroplast membrane permeability (Evans et al., 2009; Tosens et al., 2012). However, a verdict on the co-regulation of both remains to be presented. Centritto et al. (2003) and Warren (2008) argued that a linear relationship between g_m_ and g_s_ is not ubiquitous but rather differs among species and levels of water stress. On the other hand, studies by Loreto et al. (1992), Flexas et al. (2002, 2008), Ethier et al. (2006), and Perez-Martin et al. (2009) show a strong co-regulation between g_m_ and g_s_. The results from our experiment confirmed a co-regulation of both limiting components of the CO_2_ diffusion pathway (Figure 6). An interesting finding was additionally observed if plant sensitivity studied under drought. The current works highlighted that both g_m_ and g_s_ operate sequentially rather than in parallel, and that the mechanisms of their co-regulation in wheat are still not fully clear. However, the responses of g_m_ and g_s_ to environmental stimuli have recently been studied intensively (Barbour et al., 2010; Easlon et al., 2014).

The g_m_ in our experiment responded more rapidly than g_s_, as also suggested by Flexas et al. (2008), Bunce et al. (Bunce, 2009), and Keenan et al. (2010), and their mutual dependence was found to be statistically significant (*r*^2^ = 0.77). Based on our results, we support the suggestions of Flexas et al. (2006, 2007) and Warren et al. (Warren, 2008) in that these two parameters of the CO_2_ diffusion pathway in photosynthesizing leaves are dependent on each other. This work has also shown that the relationship is highly variable in many species and could be affected by a variety of environmental factors.

Although the increase in stomatal (L_S_) and mesophyll (L_M_) limitations to photosynthesis as a result of water scarcity is quite well-documented, processes linked to these phenomena are still a matter of debate (Flexas and Medrano, 2002). Restricted CO_2_ diffusion from the surrounding atmosphere to chloroplasts is a common response to water deficit and is caused by limiting factors to photosynthesis even under mild stress conditions (Roupsard et al., 1996; Grassi and Magnani, 2005; Chaves et al., 2009). To study the impact of drought and to demonstrate which limits of photosynthesis dominate, A_N_–C_i_ response curve analyses are often used (Ni and Pallardy, 2009).

In our analysis of L_S_ and L_M_ under progressive drought stress (Figure 7), genotype differences in these parameters were observed. A variety of differences could be dependent on both the intensity and duration of stress, as well as different abilities to respond to water shortage (Grassi and Magnani, 2005). Under the initial water stress, L_S_ dominated over L_M_ in the Pehlivan genotype (Figure 6). Furthermore, as the water stress developed, L_M_ increased and became crucial. The reason was simply the decline of g_m_, which was caused by the reduced CO_2_ concentration within chloroplasts. However, L_S_ has not yet been distinguished at this point in comparison with L_M_. This was caused by only a slight change in the intercellular CO_2_ concentration (C_*i*_), as also found by Lawlor and Cornic (2002). Of course, L_S_ increased as well. However, its development was less sufficient compared to L_M_. The same result for the function of L_M_ was reported in the studies by Galmés et al. (2007) and Tosens et al. (2012).

Our photosynthesis limitation analysis showed that the dynamics of the changes in L_S_ and L_M_ were different in genotypes GK Forrás, Piopi-4 and Šamorínska (Figures 7A,C,D). Since the beginning of dehydration, L_M_ and L_S_ have increased concurrently, as was also observed by Martin-Ruiz and Torres (1992). However, L_M_ began to dominate immediately from the first day of dehydration, as was also observed in the work of Delfine et al. (2001). They argued that the high values of L_M_ indicate the reduction of g_m_ and that the increase in L_M_ is responsible for the impairment of plant metabolism. L_M_ values above 80% were also demonstrated by Gallé et al. (2009) in tobacco plants. Other studies (Escalona et al., 1999) observed significant increases in L_S_ and L_M_ at the same time of a stress. Finally, we obtained similar results as documented in the studies of Flexas et al. (2014), Limousin et al. (2010), Misson et al. (2010), and StPaul et al. (2012), which stated that L_S_ is a more important factor during early drought events; however, under severe water stress, L_M_ dominates over L_S_ and primarily limits wheat photosynthesis.

## Conclusions

The present results show a significant inter-genotypic variability in wheat photosynthetic responses to a long-term progressive drought, as studied in four selected wheat genotypes of different geographical origins and breeding chronology. Our study demonstrated the effect of low water availability in plants on g_m_ inhibition. Drought clearly reduced g_m_ during long-term progressive dehydration in all wheat genotypes. The results show that g_m_ is co-regulated with g_s_ with their strong effect on A_N_ regulation. Interestingly, g_m_ is a genotypic variable not only for the conditions of drought but also for well-watered plant conditions. Therefore, we offer reliable evidence of a crucial role for g_m_ in the regulation of CO_2_ assimilation under both well-watered and drought conditions. We also demonstrated a rapid response of g_m_ to short-term C_i_ changes with significant genotypic variability under WW conditions. However, this response is significantly reduced without any genotypic effect during prolonged drought. For future research, we suggest the study of leaf anatomical traits linked to the limitations of photosynthesis together with an evaluation of plant photosynthetic parameters. It has been hypothetized, and in some individual works already demonstrated, that the differences in leaf anatomy may have a rather significant influence on the CO_2_ diffusion within the leaf mesophyll and on the whole leaf photosynthetic performance. In summary, the present results with wheat are statistically remarkable, and they contribute to the general knowledge of the regulation of leaf photosynthesis under periods of water scarcity by the mesophyll and stomata.

## Author contributions

HS, KO, MB designed the experiment and revised the paper; MK, MZ performed the experiment; KO, PS, MZ, MK analyzed the data and finished the original paper.

### Conflict of interest statement

The authors declare that the research was conducted in the absence of any commercial or financial relationships that could be construed as a potential conflict of interest. The reviewer JL declared a shared affiliation, though no other collaboration, with several of the authors HS, MB to the handling Editor, who ensured that the process nevertheless met the standards of a fair and objective review.

## Abbreviations

Γ^*^: chloroplastic CO_2_ compensation point
Φ_PSII_: actual photochemical efficiency of photosystem II
A_N_: net CO_2_ assimilation rate
C_a_: ambient CO_2_ concentration
C_c_: CO_2_ concentration at the carboxylation site of Rubisco
C_i_: CO_2_ concentration in sub-stomatal cavities
g_m_: mesophyll conductance
g_s_: stomatal conductance
J_f_: electron transport rate
MS: mild water stress
PPFD: photosynthetically active photon flux density
R_d_: day respiration rate
RWC: relative water content
SS: severe water stress
Vc_max_: maximal *in vivo* carboxylation activity of Rubisco
WUEi: intrinsic water use efficiency
WW: well-watered conditions
c.v.: coefficient of variability.
